# DNA hypermethyation and silencing of *PITX1* correlated with advanced stage and poor postoperative prognosis of esophageal squamous cell carcinoma

**DOI:** 10.18632/oncotarget.21375

**Published:** 2017-09-28

**Authors:** Takeshi Otsubo, Kazuhiko Yamada, Teruki Hagiwara, Kenshiro Oshima, Kei Iida, Koro Nishikata, Tetsuro Toyoda, Toru Igari, Kyoko Nohara, Satoshi Yamashita, Masahira Hattori, Taeko Dohi, Yuki I. Kawamura

**Affiliations:** ^1^ Department of Gastroenterology, The Research Center for Hepatitis and Immunology, Research Institute, National Center for Global Health and Medicine, Chiba, Japan; ^2^ Department of Surgery, National Center for Global Health and Medicine, Tokyo, Japan; ^3^ Department of Computational Biology and Medical Sciences, Graduate School of Frontier Science, The University of Tokyo, Chiba, Japan; ^4^ Medical Research Support Center, Graduate School of Medicine, Kyoto University, Kyoto, Japan; ^5^ Integrated Database Unit, Advanced Center for Computing and Communication (ACCC), RIKEN, Saitama, Japan; ^6^ Pathology Division of Clinical Laboratory, National Center for Global Health and Medicine, Tokyo, Japan; ^7^ Cooperative Major in Advanced Health Science, Graduate School of Advanced Science and Engineering, Waseda University, Tokyo, Japan

**Keywords:** methylome, transcriptome, tumor suppressor gene, homeobox gene, prognosis marker

## Abstract

Esophageal squamous cell carcinoma (ESCC) is associated with the accumulation of genetic and epigenetic changes in the background mucosa. Dysregulated DNA methylation is known to lead to the inactivation of tumor suppressor genes and the activation of oncogenes. To identify the genes whose expression is perturbed by abnormal DNA methylation in ESCC, integrative transcriptomics by serial analysis of gene expression (SAGE) and methylome sequencing by methyl-DNA immunoprecipitation (MeDIP) analysis were performed. We found 159 genes with significantly decreased expression in ESCC compared to that in noncancerous esophageal mucosa. MeDIP-seq analysis identified hypermethylation in the promoter region of 56 of these genes. Using surgically resected tissues of 40 cases, we confirmed that the paired-like homeodomain 1 *(PITX1)* gene was hypermethylated in ESCC compared to that in normal tissues (*P* < 0.0001) by pyrosequencing. PITX1 overexpression in ESCC cell lines inhibited cell growth and colony formation, whereas PITX1 knockdown accelerated cell growth. A PITX1-transfected ESCC cell line, KYSE30, formed smaller tumors in nude mice than in mock-transfected cells. Hypermethylation of *PITX1* was associated with tumor depth (*P* = 0.0011) and advanced tumor stage (*P* = 0.0052) and predicted poor survival in ESCC (hazard ratio, 0.1538; 95% confidence interval, 0.03159–0.7488; *P* = 0.0169). In this study, we found a novel tumor suppressor gene of ESCC, PITX1, which is silenced by DNA hypermethylation. Downregulation of PITX1 contributes to the growth and progression of ESCC. Hypermethylation of the *PITX1* in ESCC correlated with tumor progression and advanced stage cancer, and may predict a poor prognosis.

## INTRODUCTION

Esophageal squamous cell carcinoma (ESCC) is the major histological subtype of esophageal cancer and one of the most frequent fatal malignancies in the world, especially in Asian countries [1]. Environmental and lifestyle factors, such as smoking and alcohol consumption, are the risk factors for ESCC in Western countries [2], and the consumption of hot beverages has been shown to be a major risk factor in the East [3]. As with many other types of cancers, exposure of the esophageal epithelial mucosa to such conditions induces genetic mutations and epigenetic changes. These changes accumulate, induce mucosal dysplasia, and eventually, invasive squamous cell carcinoma.

CpG island hypermethylation occurs frequently in cancer cells and is associated with transcriptional repression and subsequent reduction or loss of gene function [4]. These events result in the loss of a benign phenotype and acquisition of malignant properties [5]. A previous global study using bead arrays to detect promoter-DNA methylation in ESCC demonstrated that a total of 37 CpG sites were differentially methylated between esophageal squamous cell tumors and normal mucosa [6]. The hypermethylation of some genes, such as *APC*, *FHIT*, *PGP9.5*, and *TFLC1*, is related to certain clinicopathological features of ESCC, including poor prognosis and treatment responses [7]. These findings suggest a biological significance for differentially methylated genes in ESCC. Recently, we reported that increased DNA methylation on the promoter of periplakin (*PPL*), a desmosomal protein, significantly altered ESCC stratification and led to a metastatic phenotype [8]. *PPL* had never been reported as a hypermethylated gene associated with ESCC, possibly due to the high methylation status of the PPL promoter region in the surrounding noncancerous mucosa. These results encouraged us to perform a genome-wide DNA methylation analysis, combined with a complete expression profile, to identify epigenetically regulated molecular players in ESCC.

With the advent of next-generation sequencing technologies, methyl-DNA immunoprecipitation (MeDIP) analysis has become a powerful tool for comprehensive characterization of DNA methylation status in coding and non-coding regions of the genome. In this study, we assessed the genes displayed abnormal methylation in its promoter region in ESCC at a global level. We used MeDIP-seq and integrated the methylome profile with data from transcriptome sequencing by serial analysis of gene expression (SAGE) to find candidate genes with altered regulation and DNA methylation status. These candidates may be involved in the development and progression of ESCC. Herein, we found that paired-like homeodomain 1 (PITX1) was markedly suppressed by hypermethylation on the promoter in ESCC samples. We restored *PITX1* expression in ESCC cell lines and found that PITX1 functions as a tumor suppressor in ESCC progression both *in vitro* and *in vivo*. Finally, we demonstrated for the first time that hypermethylation of *PITX1* was a poor prognostic factor for patients with ESCC.

## RESULTS

### Differentially expressed genes (DEGs) in ESCC and correlation with abnormal promoter methylation

To uncover genes with disturbed expression and abnormal methylation in the promoter region, transcriptome and methylome analyses were performed and integrated. First, to search for differentially expressed genes (DEGs) between normal esophageal mucosa and ESCC, serial analysis of gene expression (SAGE)-seq was performed. Result of transcriptome is uploaded at GSE71411. In total, 59 genes had significantly increased expression in ESCC relative to normal mucosa (WAD score cutoff value = 1.7). Additionally, 159 genes had significantly decreased expression in ESCC. Next, to search for differentially methylated regions (DMRs) between normal esophageal mucosa and ESCC, methyl-DNA immunoprecipitation (MeDIP)-seq was performed. Result of methylome is uploaded at GSE71410. Hypermethylated regions in ESCC relative to normal mucosa totaled 123,020, and 79,436 hypo-methylated regions were found (log-fold change (logFC) cutoff value = 2). To integrate these data, the distance of each DMR from the transcriptional start site (TSS) of a gene was calculated, and a gene was designated as differently methylated if it had DMR from -10 kb to +2 kb from TSS. Integrated analysis of DEGs and DMRs identified 5 of 59 over-expressed genes with promoter hypo-methylation (Table 1) and 56 of 159 downregulated genes with promoter hypermethylation (Table 2) in ESCC.

**Table 1:** Up-regulated genes with promoter hypo-methylation in ESCC

Gene Symbol	Gene ID	WAD Score	Location of CpG island^*^
LOXL2	NM_002318	-1.8686	Promoter
MAGEA2	NM_001282501	-4.071	No
MAGEA2B	NM_153488	-4.8095	No
KHDC1L	NM_001126063	-2.6755	No
TREM2	NM_018965	-3.4774	No

^*^‘Promoter’ means that CpG island was located the promoter region of indicated genes and hypo-methylated in ESCC as compared in normal esophageal mucosa. ‘No’ means that indicated genes have no CpG island.

**Table 2:** Down-regulated genes with promoter hyper-methylation in ESCC

Gene Symbol	Gene ID	WAD Score	Location of CpG island^*^
SCGB3A1	NM_052863	-2.6608	Promoter, Gene body
NCCRP1	NM_001001414	-2.6962	Promoter, Gene body
FKBP1A-SDCBP2	NR_037661	-2.2573	Promoter, Gene body
RHCG	NM_016321	-2.4623	Promoter, Gene body
PPL	NM_002705	-1.9138	Promoter, Gene body
PITX1	NM_002653	-1.7584	Promoter, Gene body
PRSS27	NM_031948	-4.071	Promoter
MAL	NM_002371	-4.8095	Promoter
CES1	NM_001025194	-2.6755	Promoter
MYH11	NM_002474	-3.4774	Promoter
VSIG10L	NM_001163922	-1.8686	Promoter
GSTO2	NM_183239	-2.3154	Promoter
GPX3	NM_002084	-2.4782	Promoter
FAM174B	NM_207446	-2.2616	Promoter
KRT7	NM_005556	-1.7158	Promoter
SCNN1B	NM_000336	-1.8062	Promoter
CST6	NM_001323	-1.7807	Promoter
C15orf48	NM_032413	-2.3005	Promoter
RANBP9	NM_005493	-2.2885	Promoter
SPINT1	NM_181642	-1.7312	Promoter
C9orf16	NM_024112	-1.8787	Promoter
HOPX	NM_001145459	-2.1194	Promoter
UBXN1	NM_001286077	-1.7014	Promoter
TMEM141	NM_032928	-1.8507	Promoter
CUL3	NM_003590	-1.706	Promoter
ECHS1	NM_004092	-1.8793	Promoter
FAM129B	NM_022833	-1.7615	Promoter
FUT3	NM_000149	-2.3663	Gene body
UGT1A10	NM_019075	-1.8534	Gene body
UGT1A3	NM_019093	-1.8502	Gene body
UGT1A6	NM_001072	-1.8502	Gene body
UGT1A7	NM_019077	-1.8626	Gene body
KRT78	NM_173352	-4.8046	No
IVL	NM_005547	-3.5593	No
CRCT1	NM_019060	-3.8779	No
SPRR2A	NM_005988	-3.8847	No
KRT4	NM_002272	-4.5689	No
KRT13	NM_153490	-4.2105	No
A2ML1	NM_144670	-2.0995	No
C2orf54	NM_001085437	-2.2958	No
ZG16B	NM_145252	-2.3464	No
CRISP3	NM_001190986	-2.3873	No
SPRR3	NM_001097589	-3.8629	No
KLK13	NM_015596	-1.9099	No
SPRR1A	NM_001199828	-2.8358	No
GBP6	NM_198460	-2.5643	No
MGST1	NM_001260511	-1.775	No
ACTG2	NM_001615	-2.424	No
RPSAP9	NR_026890	-1.884	No
MFAP4	NM_002404	-1.7146	No
LMO7	NM_015842	-1.9632	No
DAPL1	NM_001017920	-1.755	No
EMP1	NM_001423	-2.5968	No
ALDH3B2	NM_000695	-1.833	No
CSTA	NM_005213	-2.8041	No
SEPP1	NM_001085486	-1.7591	No

^*^‘Promoter, Gene body’ means that CpG islands were located in the promoter region and gene body of indicated genes but ESCC-specific hyper-methylation was observed only in their promoters. ‘Promoter’ means that CpG island was located the promoter region of indicated genes and hyper-methylated in ESCC as compared in normal esophageal mucosa. ‘Gene body’ means that CpG island was located only in the gene body of indicated genes and ESCC-specific hyper-methylation was observed in their promoters. ‘No’ means that indicated genes have no CpG island.

**Figure 1:**
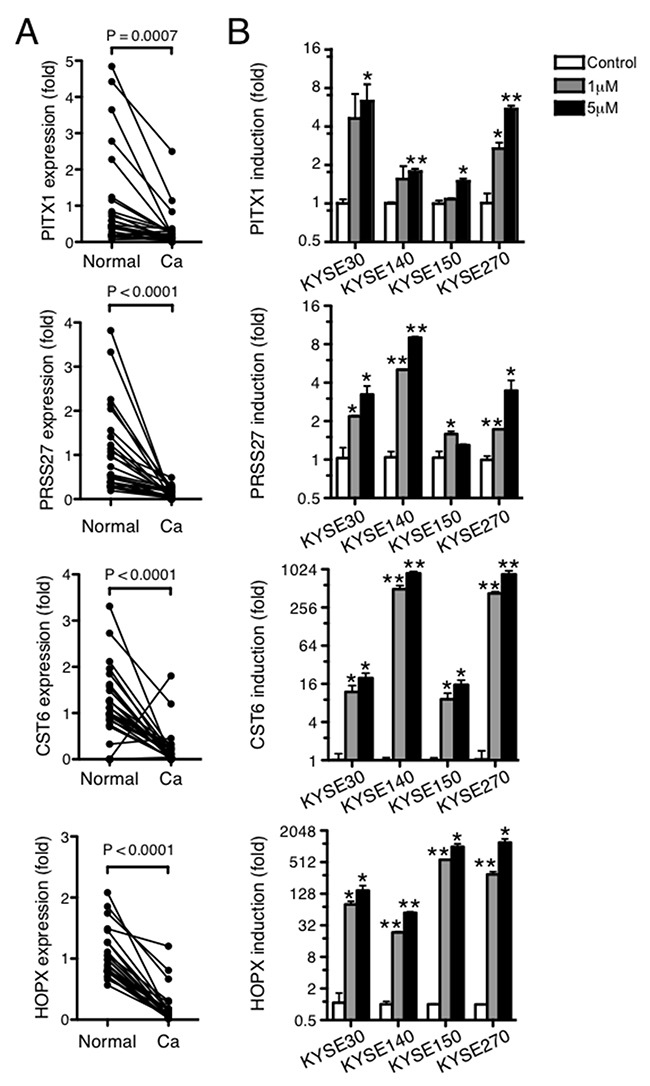
The expression of identified genes by integrative transcriptome and methylome analyses is silenced by DNA methylation in ESCC **(A)** Transcript levels of *PITX1*, *PRSS27*, *CST6*, and *HOPX* were determined by qRT-PCR in duplicate for 32 ESCC samples. Expression levels are reported relative to the mean levels of normal tissues. **(B)** The mRNA induction of *PITX1*, *PRSS27*, *CST6*, and *HOPX* in ESCC cell lines after treatment with 1 μM or 5 μM of 5-aza-dC. Data are reported as the fold increase in induction relative to that in untreated cells and shown as mean ± SD of assays performed in duplicate. ^*^P<0.05, ^**^P<0.01.

Among the cancer-specific hypermethylated genes, *SCGB3A1* [9] and *PPL* [8] were already reported to have hypermethylation at its promoter, leading to reduced expression in ESCC. PITX1 [10–15], CST6 [16, 17] and HOPX [18–21] were reported their downregulation in various types of human cancer, and PRSS27 [22] was known to be highly expressed in nonkeratinizing stratified squamous epithelia of human esophagus and its dysregulation was supposed to be related to characteristics of carcinoma. Thus, we initially started to evaluate results from the SAGE-seq analysis by quantitative RT-PCR using paired normal and ESCC frozen tissue samples from 32 patients on these 4 genes. The expression of these genes was significantly decreased in the cancer tissues compared with those in matched normal mucosa (Figure 1A). To confirm the results of MeDIP-seq, DNA hypermethylation-induced gene silencing was also examined in the ESCC cell lines. Endogenous mRNA expression levels were determined in four ESCC cell lines (KYSE30, KYSE140, KYSE150 and KYSE270) and compared with the levels in cells treated with 5-aza-2′-deoxycytidine (5-aza-dC), an inhibitor of DNA methyltransferase. In all cell lines, treatment with 5-aza-dC significantly increased selected gene expression (Figure 1B). These results indicate that PITX1, PRSS27, CST6, and HOPX selected from our integrative analysis are likely the targets of aberrant DNA hypermethylation, and DNA hypermethylation likely downregulated expression in ESCC. Among the differentially methylated genes uncovered here, *PITX1* was selected for further analysis, because we were interested in its previously reported function as negative regulator for the telomerase reverse transcriptase (TERT), which plays an important role in the cell growth [23].

### PITX1 protein expression was lower in ESCC than in normal esophageal mucosa

**Figure 2:**
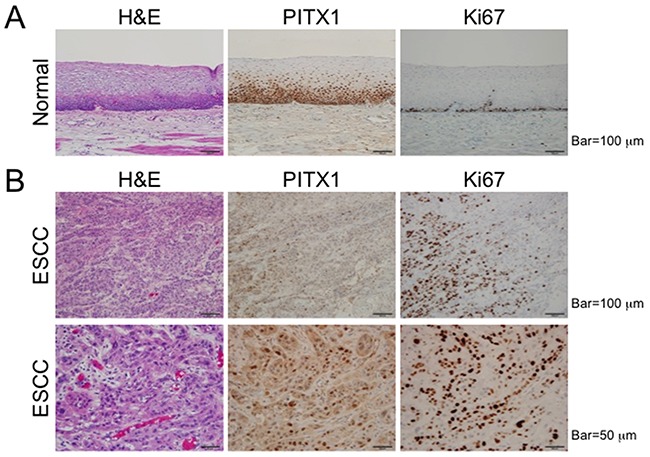
Decreased expression of PITX1 in ESCC Representative images produced from formalin-fixed, paraffin-embedded samples of normal mucosa **(A)** and ESCC **(B)** stained with H&E, anti-PITX1, or anti-Ki67 antibodies.

To investigate the protein expression and localization of PITX1 in the normal esophageal mucosa or ESCC, immunohistochemical analyses were performed. In the normal mucosa, PITX1 protein was detected in the nuclei of a majority of squamous cells, including the basal cell layer and spinous cells in the suprabasal cell layer. Cells in several stratified layers close to surface were not positive for PITX1 protein expression (Figure 2A). Proliferating Ki67^+^ cells were found only in basal cell layer, and all of these cells expressed PITX1. In ESCC samples, the expression of PITX1 was dramatically decreased, while the expression of Ki67 had increased (Figure 2B). The percentage of PITX1- and Ki67-expressing cells was quantified. More than 80 % of nuclei in the normal mucosal layer expressed PITX1; however, the average percentage of PITX1^+^ cells in ESCC samples examined was 38.3%. The average percentage of Ki67^+^ cells of ESCC (67.0%) was higher than that of normal tissues (less than 10%). Double staining with anti-PITX1 and anti-Ki67 antibodies was difficult because the conditions for antigen retrieval for each protein were markedly different; however, these results indicated the emergence of ESCC-specific, PITX1^-^Ki67^+^ proliferating cells, which were never detected in the normal tissues. In other words, the loss of *PITX1* may be associated with cancer cell proliferation.

### Ectopic PITX1 expression inhibited cell growth *in vitro* and *in vivo*

**Figure 3:**
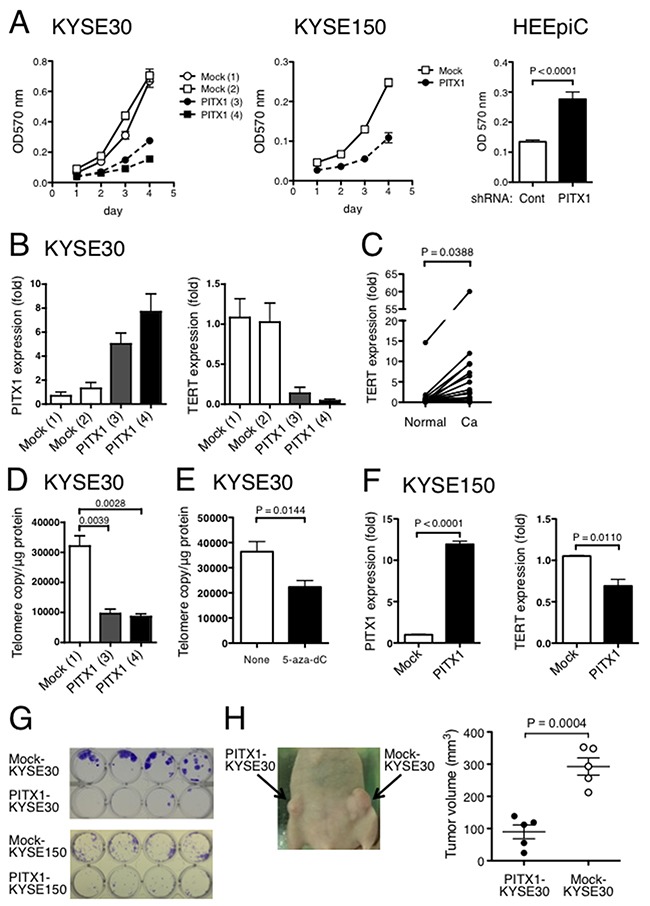
Ectopic expression of PITX1 suppressed ESCC cell growth *in vitro* and *in vivo* **(A)** Growth curves of *PITX1*-transfected and mock-transfected KYSE30 clones and KYSE150 stable transfectants as determined by MTT assay (left). HEEpiC cells were transiently transfected with control or PITX1-shRNA. Forty-eight hours after transfection, cell growth was assessed by MTT assay (right). Data are shown as mean ± SD of assays performed in triplicate. **(B)** *PITX1* (left) and *TERT* (right) mRNA levels in 2 mock-transfected and 2 *PITX1*-transfected clones of KYSE30 cells. Numbers in parentheses indicate clone names. Data are shown as mean ± SD of assays performed in triplicate. **(C)** Increased expression of *TERT* in ESCC. *TERT* transcript levels were measured in paired samples from 32 ESCC samples by RT-PCR. Data are reported as expression relative to the mean level in normal tissues. **(D)** Telomerase activity in *PITX1*-transfected and mock-transfected clones of KYSE30 cells. Data are shown as mean ± SD of assays performed in triplicate. **(E)** Inhibition of telomerase activity in KYSE30 cells after treatment with 5 μM of 5-aza-dC. Data are shown as mean ± SD, assays were repeated five times. **(F)** *PITX1* (left) and *TERT* (right) mRNA levels in stably mock-transfected and *PITX1*-transfected KYSE150 cells. Data are shown as mean ± SD of assays performed in triplicate. **(G)** Colony formation of stably *PITX1*-transfected and mock-transfected KYSE30 and KYSE150 cells. Cells were cultured for 2 weeks. Colonies were visualized by crystal violet staining. **(H)** Representative images of subcutaneous xenografts (left). Nude mice were subcutaneously inoculated with *PITX1*-transfected KYSE30 cells in the right flank and mock-transfected KYSE30 cells in the left flank. Three weeks after inoculation, tumor volumes were measured.

To clarify the significance of PITX1 defects in ESCC, *PITX1*-transfected and mock-transfected ESCC cell lines, KYSE30 and KYSE150, were prepared and examined for the effect of ectopic *PITX1* expression on their growth. In both ESCC cell lines used, the ectopic expression of *PITX1* suppressed cell growth compared with mock transfectants (Figure 3A). Two clones of stably *PITX1*-transfected KYSE30 cells expressed *PITX1* mRNA at different levels: clones 3 and 4 expressed *PITX1* 5- and 8-fold higher than the mock clones (clone 1 or 2), respectively. In *PITX1*-transfected KYSE30 cells, the growth inhibitory effect of *PITX1* appeared to be dose-dependent on mRNA level (Figure 3A and 3B). Furthermore, human normal epithelial cell HEEpiC was treated with control or PITX1-shRNA and examined for the effect of *PITX1* suppression on its growth. Forty-eight hours after transfection, the growth of PITX1-shRNA treated cells was higher than that of control shRNA-treated cells (Figure 3A). Since *PITX1* has been reported as negative regulator for TERT, we examined the expression levels of *TERT* by quantitative RT-PCR, and then compared them between ESCC and paired normal tissues. In human tissue samples, expression levels of *TERT* in the cancer tissues were significantly higher than those in matched normal mucosa (*P* = 0.0388; Figure 3C). We next examined for the effect of ectopic *PITX1* expression on the *TERT* transcription. Two clones of stably *PITX1*-transfected KYSE30 cells exhibited a significant decrease in *TERT* mRNA expression (Figure 3B). In agreement with the results on *TERT* expression, the ectopic expression of *PITX1* inhibited telomerase activity in both *PITX1*-transfected clones (Figure 3D). In addition, treatment of KYSE30 cells with 5-aza-dC resulted in a 0.6-fold decrease in telomerase activity (Figure 3E), concomitant with the induction of *PITX1* expression (Figure 1B). Stably *PITX1*-transfected KYSE150 cells also exhibited significantly decreased expression of *TERT* mRNA as compared with the mock-transfected KYSE150 cells (Figure 3F). In a colony formation assay, *PITX1* transfectants of both ESCC cells lines failed to form colonies, while mock transfectants efficiently formed colonies (Figure 3G). Finally, *PITX1*- and mock-transfected KYSE30 cells were used in a xenograft model of tumor formation in BALB/c nude mice. *PITX1* transfectants formed smaller tumors than mock transfectants (Figure 3H). Collectively, these results demonstrate that expression of *PITX1* inhibits cell growth of ESCC *in vitro* and *in vivo*.

### DNA hypermethylation of *PITX1* was associated with advanced tumor stage and poor survival in ESCC

**Figure 4:**
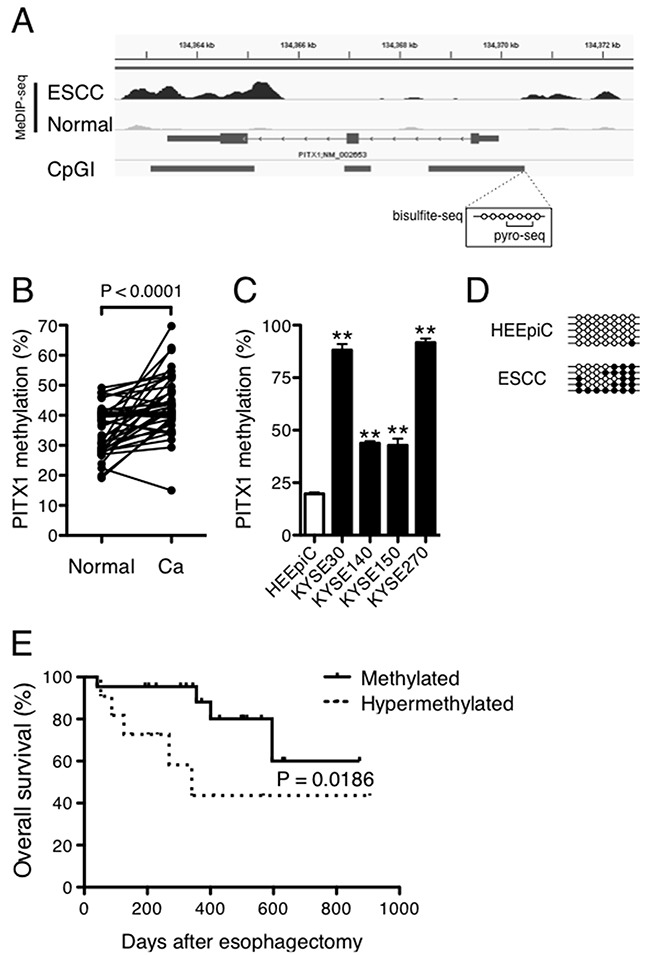
DNA hypermethylation of *PITX1* is correlated with a poor prognosis of ESCC **(A)** The MeDIP-seq signal tracks at the *PITX1* locus in ESCC and normal mucosa. The Y-axis represents the normalized MeDIP-seq-tags. The gray bars at the bottom show the positions of the CpG islands, and the circles on the lines in the box represent the CpGs analyzed by pyrosequencing and bisulfite sequencing. **(B)** DNA methylation of *PITX1* was determined by pyrosequencing of paired samples from 40 patients with ESCC. **(C)** DNA methylation of *PITX1* was determined by pyrosequencing of HEEpiC and ESCC cell lines. Data are shown as mean ± SD of assays performed in triplicate. ^**^P < 0.01, as compared to HEEpiC. **(D)** Methylation status of individual CpG residues in the *PITX1* gene in HEEpiC cells and ESCC sample assessed by bisulfite sequencing. Bisulfite-PCR products cloned into the pCR4-TOPO vector were randomly selected for sequencing. Each line indicates an independent clone of bisulfite-PCR products containing 7 consecutive CpGs. Filled circles on the lines for each clone appear only when CpGs are methylated, and open circles appear only when CpGs are unmethylated. **(E)** Kaplan–Meier curves of ESCC patient survival. There was a significant difference in survival between the *PITX1*-hypermethylated and *PITX1*-methylated groups.

MeDIP-seq analysis demonstrated hypermethylation at the *PITX1* locus in ESCC compared to that in normal mucosa (Figure 4A). Finally, to understand the significance of hypermethylation in the *PITX1* gene, the DNA methylation levels of CpG islands were determined for the PITX1 promoter using pyrosequencing, and then they were compared between ESCC and paired normal tissues. The average methylation level was 44.9 ± 10.7% in the tumor samples, compared with 35.2 ± 7.9% in the normal mucosa samples. A paired *t*-test indicated that the tumor DNA was significantly more methylated than that in the normal tissues (Figure 4B). Methylation statuses of *PITX1* in ESCC cell lines were also assessed by pyrosequencing. Hypermethylation of *PITX1* was observed in all ESCC cell lines as compared to that in human normal epithelial cell HEEpiC (Figure 4C). Because pyrosequencing reflects the methylation status of only 3 adjoining CpG motifs, the PCR products, containing 7 CpGs as shown in Figure 4A, were subjected to bisulfite sequencing. As expected, methylated CpGs were hardly seen in the HEEpiC cells, whereas it was clearly evident that the upstream region of the *PITX1* gene was frequently hypermethylated in ESCC tissues (Figure 4D). These results suggest that the loss of *PITX1* by DNA hypermethylation is associated with cancer cell proliferation. To further clarify the clinical relevance of *PITX1* hypermethylation in ESCC, we analyzed the methylation status of the *PITX1* gene and clinicopathologic characteristics of 40 patients with ESCC. We classified the samples into two groups: 1) percentage of methylated *PITX1* DNA was ≥50% by pyrosequencing (hypermethylated) and 2) methylated DNA was less than 50 % (methylated). Of the 40 primary tumors studied, 13 were classified as hypermethylated (Table 3). Univariate analysis revealed no difference between the hypermethylated and methylated groups with respect to gender, tumor location, lymph node metastasis, or distant metastasis. However, there were significant differences between patients in the hypermethylated and methylated groups with respect to age (*P* = 0.0388), tumor depth (*P* = 0.0017), and tumor stage (*P* = 0.0053).

**Table 3:** Clinicopathological features of ESCC with or without methylation of *PITX1*

Characteristics	Total	Number of patients (%)		
		*PITX1*		
		Hypermethylated	Methylated	P-value
Number of patients	40	13 (32.5)	27 (67.5)	
Mean age ± SD (years)		63.38 ± 12.26	70.74 ± 10.12	0.0388
Sex				
Male	31 (77.5)	8 (61.5)	23 (81.2)	0.0935
Female	9 (22.5)	5 (38.5)	4 (14.8)	
pT classification				
T1	13 (32.5)	2 (15.4)	11 (40.7)	0.0017
T2	6 (15.0)	1 (7.7)	5 (18.5)	
T3	15 (37.5)	4 (30.8)	11 (40.7)	
T4	6 (15.0)	6 (46.1)	0 (0.0)	
pN classification				
N0	18 (45.0)	5 (38.4)	13 (48.2)	0.1507
N1	12 (30.0)	2 (15.4)	10 (37.0)	
N2	6 (15.0)	4 (30.8)	2 (7.4)	
N3	4 (10.0)	2 (15.4)	2 (7.4)	
pM classification				
M0	37 (92.5)	13 (100.0)	24 (88.9)	0.2114
M1	3 (7.5)	0 (0.0)	3 (11.1)	
Cancer stage				
I	13 (32.5)	3 (23.1)	10 (37.0)	0.0053
II	11 (27.5)	1 (7.7)	10 (37.0)	
III	13 (32.5)	9 (69.2)	4 (14.8)	
IV	2 (5.0)	0 (0.0)	3 (11.1)	
Tumor location				
Upper thoracic	12 (30.0)	3 (23.1)	9 (33.4)	0.5981
Middle thoracic	17 (42.5)	7 (53.8)	10 (37.0)	
Lower thoracic	11 (27.5)	3 (23.1)	8 (29.6)	

We then applied univariate analysis to evaluate the association between the methylation status of the *PITX1* gene and patient prognosis. Kaplan–Meier curve analysis showed a significant difference between patients in the hypermethylated and methylated groups with respect to overall survival rate (hazard ratio, 0.1538; 95% confidence interval, 0.03159–0.7488; *P* = 0.0169; Figure 4B). These results suggest that the DNA hypermethylation of *PITX1* is associated with cancer progression and predicts poor postoperative survival in ESCC.

## DISCUSSION

In this study, we demonstrated that the nuclear expression of PITX1, a member of the RIEG/PITX1 homeobox family, was suppressed in ESCC. Although downregulation of *PITX1* was reported in various types of human cancer, including colon cancer cell lines, prostate and bladder tumors [10], lung [11], and gastric cancers [12], Barrett’s-adenocarcinoma [13], oral tumors [14] and malignant melanoma [15], this report is the first to show a role for PITX1 in ESCC. Furthermore, these results point to a mechanism of *PITX1* downregulation through elevated promoter methylation levels. Hypermethylation of *PITX1* clearly correlated with advanced tumor size, advanced tumor stage, and a poor prognosis, suggesting the possibility of the methylation status of *PITX1* as prognostic markers of ESCC. Protein localization of PITX1 in the esophageal benign and malignant mucosa, and its function of regulating the growth of ESCC, were also illustrated.

The results presented here clearly demonstrate that PITX1 is one of the targets of aberrant DNA hypermethylation in ESCC. A previous global study using bead arrays to detect promoter-DNA methylation uncovered 37 CpG sites that were differentially methylated between esophageal squamous cell tumors and normal mucosa [6]. PITX1, however, was not specifically listed in that report or other previous reports investigating hypermethylated genes in ESCC. This difference may be the result of the selected methodology for methylome analysis. In the method using bead arrays, bisulfite-converted genomic DNA is hybridized to probes designed against selected loci. Thus, by using bead arrays, DNA methylation patterns can be interrogated in a locus-targeted manner. In contrast, MeDIP-seq can detect DMRs across the entire genome by sequencing of DNA captured by the MBD2 protein, resulting in a more comprehensive analysis of the entire genome. Another reason that PITX1 was not previously detected may be the high methylation status of the PITX1 promoter region in the normal/background mucosa; the lowest level of the PITX1 promoter methylation in all the normal tissue samples was 19% in our study. Although the difference was not dramatic, there was a statistically significant increase in the methylation levels on PITX1 in tumors. Thus, this method had advantage to detect ESCC-related changes those were not “all-or-none” type difference but significant additional changes to background mucosa. In fact, the presence of aberrant DNA hypermethylation in noncancerous esophageal mucosa of ESCC cases was reported in association with smoking history [24]. As shown in the case of PITX1, modest but significant additional DNA methylation to the esophageal mucosa may trigger the development of ESCC. Combined with our previous publications, we have now reported two new target genes of increased promoter-DNA methylation in ESCC: PITX1 and PPL [8]. In addition to these genes, our integrated analysis of DNA methylome and transcriptome identified 59 genes. Expression patterns of these genes may be disrupted by abnormal methylation in ESCC. These results suggest that sequence-based methylome analysis is an effective tool for identifying novel methylation hotspots. This tool is especially powerful for the identification of genes with background methylation levels that are higher than typically observed, possibly due to exposure to various environmental risk factors.

Another noteworthy observation is that DNA hypermethylation of PITX1 correlated with advanced tumor size, advanced tumor stage, and a poor postoperative prognosis. ESCC is characterized by high malignancy and a poor prognosis. Although PITX1 methylation may correlate with total epigenetic alteration of many genes, which is associated with the phenotypes of clinically advanced tumor cells, our studies clearly demonstrate that PITX1 methylation status of the tumor could be used as a clinical marker to predict prognoses. Multimodal therapy has been performed for ESCC, in combination of chemotherapy, radiation therapy and surgery. Since surgery for ESCC is the most invasive remedy, treatment without operation is sometime chosen. However, currently, there is no marker to support the selection of the most appropriate combination of treatment. At least, PITX1 methylation status of the tumor could be one of the useful references for clinical decision making of invasive surgery. In this study, we have not analyzed the relationships between preoperative chemotherapy or irradiation efficacy and PITX1 methylation status. Further studies will refine the value of evaluation of PITX1 methylation status as a clinical prognostic marker of ESCC.

One major tumor-suppressive mechanism is programmed cell death; induced by the shortening of telomeres through suppressed telomerase activity [25]. Previously, PITX1 was identified as a *TERT* suppressor through the direct binding of the PITX1 protein to the *TERT* promoter [23]. In addition to canonical function of telomere length regulation, *TERT* acts as a transcriptional modulator of Wnt/β-catenin and nuclear factor-κB (NF-κB) signaling pathways. In ESCC, TERT-mediated activation of the Wnt and NF-κB signaling pathways and subsequent enhanced expression of target genes may play a direct role in facilitating cancer-promoting functions, such as proliferation and resistance to apoptosis [26, 27]. PITX1 is identified as a negative regulator of the RAS pathway through the RAS protein activator like 1 (*RASAL1*) gene, a member of the RAS GTPase-activating protein family [10]. Because activation of the RAS/mitogen-activated protein kinase (MAPK) pathway contributes to the tumorigenesis and progression of ESCC and PITX1 has been identified as a negative regulator of the RAS pathway via the downregulation of RASAL1 [10], the epigenetic silencing of *PITX1* may result in the activation of the RAS/MAPK pathway, leading to the hyperproliferation of the esophageal epithelium. These molecular mechanisms may explain the findings that forced expression of PITX1 and knockdown of PITX1inhibited and accelerated cell growth, respectively. Overall, these studies support our *in vitro* and *in vivo* results that *PITX1* is a tumor suppressor gene in ESCC.

Thus, our results are the first to indicate that *PITX1* functions as a tumor suppressor gene in ESCC, and that hypermethylation of its promoter serves as a novel biomarker for predicting prognosis in ESCC.

## MATERIALS AND METHODS

### Patients

Paired ESCC and normal tissue specimens were obtained from 40 patients who had undergone esophagectomy or esophagogastrectomy with confirmed diagnosis of ESCC. The patients were selected from those who underwent surgery between January 2013 and August 2015 at the National Center for Global Health and Medicine (NCGM). Quantitative PCR for expression analysis was performed using 32 paired frozen samples from these patients. This study was approved by the research ethics committees of the NCGM (121 or 1484), and informed consent was obtained from all the patients before the samples were collected.

The characteristics of ESCC are summarized in Table 3. Among the 40 paired ESCC samples examined in this study, paired specimens from a 62-year-old man with ESCC at pT3, pN2, and pM0 (stage IIIB) were subjected to methylome analysis and transcriptome analysis using a next-generation sequencing (NGS) system.

### Cell lines and culture

Human ESCC cell lines (KYSE140, KYSE150, and KYSE270) were purchased from the Japanese Collection of Research Bioresources Cell Bank (Osaka, Japan). KYSE30 was obtained from HPA Culture Collections. These cell lines were maintained in Ham's F12/RPMI1640 medium containing 2% FBS. Human esophageal epithelial cells (HEEpiC) were purchased from ScienCell Research Laboratories, Inc. (Carlsbad, CA) and were maintained in Epithelial Cell Medium-2 (ScienCell Research Laboratories). In some experiments, the cells were cultured in a 24-well plate at a density of 5 × 10^4^ cells/well for 18 h, and then treated with 5-aza-2′-deoxycytidine (5-aza-dC; Sigma-Aldrich, Inc., St. Louis, MO) for 72 h.

### SAGE-seq analysis

SAGE-seq libraries were generated from 5 μg of total RNA using SOLiD SAGE Kit with Barcoding Adaptor Module and multiplexed using SOLiD RNA Barcoding Kit Module 1-16 (Life Technologies, Carlsbad, CA) according to the manufacturer's instructions. Briefly, total RNA was treated with DNase I, and then bound to Dynabeads Oligo(dT) EcoP magnetic beads for cDNA synthesis. The double-stranded cDNA synthesized on the beads was digested with *Nla* III and ligated to the adaptor containing an *Eco*P15I restriction enzyme recognition site. The adapter-ligated cDNA was digested with *Eco*P15I to release the tag containing the adaptor sequence and 27 bp of unique sequence from a single transcript. Each library was amplified in a PCR reaction by 8 cycles.

### MeDIP-seq analysis

Genomic DNA was extracted from tissue specimens and sheared for the MeDIP assay. The MeDIP assay was performed by using the MethylMiner™ Methylated DNA Enrichment Kit (Life Technologies) according to the manufacturer's instructions. MeDIP specificity was confirmed by MeDIP-PCR with primer sets of the active-chromatin locus glyceraldehyde-3-phosphate dehydrogenase (*GAPDH*) and imprinted locus *H19* (data not shown). The MeDIPed DNA was prepared for NGS-seq library construction using the SOLiD Fragment Library Construction Kit and multiplexed by using SOLiD™ Fragment Library Barcoding Kit Module 1-16 (Life Technologies) according to the manufacturer's instructions. Briefly, each MeDIPed DNA was end-repaired, barcoded by adapter ligation, amplified by 15 PCR-cycles, and purified.

### Sequencing

The size of the constructed library was quantified by the 2100 Bioanalyzer system with the High Sensitivity DNA kit (Agilent Technologies, Santa Clara, CA) and the SOLiD Library TaqMan Quantitation Kit (Life Technologies). Library DNA was subjected to emulsion PCR, enrichment, and deposition onto bead according to the supplier's protocol. Finally, DNA was sequenced using the AB 5500 Genetic Analyzer (Life Technologies).

### Informatics

All short reads from the AB 5500 Genetic Analyzer were aligned to human genome version 37.3 (hg19) using the Tophat2 program with default parameters. We defined a region as a peak, if the region had more than 10 RP100M (Reads per 100 million of mapped reads) for an interval of more than 20 nucleotides [28]. The weighted average difference (WAD) score was calculated based on fold change values and weighted with degrees of expression values [28]. Sequences corresponding to differentially expressed genes (DEGs) in ESCC compared with normal mucosa were detected by a WAD score of 1.7 for SAGE-seq. Sequences corresponding to differentially methylated regions (DMRs) in ESCC relative to normal tissue were detected using Iida's methods with log-fold change (logFC) 2 for MeDIP-seq. For each gene, the distance from the transcription start site (TSS) was calculated using the NCBI Entrez annotation (hg19) as a reference. Genes having peaks of −10 kb to +2 kb from TSS were designated as DNA methylated genes.

### Reverse-transcription and quantitative PCR

Total RNA was isolated from tissues using RNA Bee RNA Isolation Reagent (Tel-Test, Inc., Friendswood, TX). After the RNA was treated with DNase I, double-stranded cDNA was synthesized using the High-Capacity cDNA Reverse Transcription Kit (Applied Biosystems, Foster City, CA). Quantitative PCR was performed using ABI TaqMan probes (Applied Biosystems) as described previously. Threshold cycle numbers were determined using the Sequence Detector software and transformed as described by the manufacturer, with glyceraldehyde-3-phosphate dehydrogenase (GAPDH) as the calibrator gene. The TaqMan Gene expression assay IDs used in this study for the genes as follows: PITX1, Hs00267528_m1; PRSS27, Hs01029754_m1; CST6, Hs01029754_m1; HOPX, Hs04188695_m1; TERT, Hs00972656_m1; GAPDH, Hs00266705_g1.

### DNA methylation analysis

We used different methods (pyrosequencing and bisulfite sequencing) to assess the PITX1 methylation status. Pyrosequencing was performed with PyroMark Gold Q24 reagents and a PyroMark Q24 pyrosequencing machine (QIAGEN, Hilden, Germany). The PCR primers used in this study were 5’-GGAATTTAGTTAGGTTGAGTGATAGTAG-3’ and 5’-AAAATCCTTAAAATCTTCCTTCTACAT-3’. The primer used for pyrosequencing was 5’-GTTGTTTTTTAATTAGTTTGGATT-3’. For bisulfite sequencing, the PCR primers used were 5’-GGGTTA GTTTAGGTAGTTTTTA-3’ and 5’-ACCATCATTTC TATCCCAATCC-3’ (231 bp). For sequencing of the bisulfite-PCR product, the DNA fragment was purified and cloned into a pCR4-TOPO vector (Invitrogen). Clones for subsequent sequencing were randomly selected.

### Ectopic expression of PITX1

To construct the expression vector of the *PITX1* gene, a DNA fragment encoding the full-length ORF (SC122251, OriGene Technologies, Rockville, MD) was digested with the restriction endonucleases *Sal*I and *Xma*I, and then the fragment was ligated into the pIRES2-EGFP vector (CLONTECH). The resulting vector was transfected into KYSE30 and KYSE150 cells using lipofectamine LTX reagent (Life Technologies). Stably transfected cells were isolated using the MoFlo^TM^ XDP Cell Sorter (Beckman Coulter, Inc.).

### Transient transfection of shRNA

To knockdown the expression of the PITX1 gene, four unique 29 mer shRNA constructs in plasmids were obtained from OriGene Technologies (TG310405). In these four 29mers (A, B, C, and D), only construct A efficiently suppressed the PITX1 expression; therefore, this construct was used. For control experiments, a non-effective 29-mer scrambled shRNA cassette in same plasmid was used. They were transfected into HEEpiC cells using lipofectamine 3000 reagent (Life Technologies) according to the manufacturer's instructions.

### Immunohistochemical analysis

Formalin-fixed, paraffin-embedded sections of surgical specimens from patients with ESCC were deparaffinized and rehydrated. Antigen retrieval was performed in 10 mM sodium citrate buffer (pH 6.0) using an autoclave for 5 min. Sections were stained with hematoxylin and eosin (H/E) or anti-PITX1 antibody (HPA008743, Sigma-Aldrich, Inc.). A diaminobenzidine staining procedure was performed using the ImmPACT^TM^ DAB peroxidase substrate kit (Vector Laboratories, Burlingame, CA), and hematoxylin was used for counterstaining. Ki67 was detected using anti-Ki67 antibody (MIB-1, Dako, Glostrup, Denmark) after antigen retrieval.

### Assay for telomerase activity

The lysates of PITX1-transfected KYSE30 cells were subjected to telomerase assay using Quantitative Telomerase Detection (QTD) Kit (Allied Biotech Inc., Vallejo, CA) according to the manufacturer's instructions. In brief, the QTD assay with real-time PCR detection system as following program; telomerase reaction at 25°C for 20 min, PCR initial activation step 95°C for 10 min, followed by 40 cycles of 30 sec each at 95°C, 60°C and 72°C. Telomere copy/microgram protein was calculated by the standard curve generated with the serial diluted control templates with protein concentration.

### Cell proliferation assays and colony formation assays

KYSE30 and KYSE150 transfectants were seeded in 96-well plates at 1000 cells per well and cell proliferation was measured by using a MTT assay kit (Nacalai tesque, Kyoto, Japan). Cell proliferation was examined every 24 h. For the colony formation assays, viable KYSE30 or KYSE 150 transfectants (100 cells) were seeded in each well in 24-well plates. After incubation for 2 weeks, colonies were stained with crystal violet.

### Cells grown as subcutaneous xenografts in athymic mice

The male BALB/c nude (nu/nu) mice were purchased from Japan SLC, Inc. (Hamamatsu, Shizuoka, JAPAN) and maintained under pathogen-free conditions in the animal facility of the National Center for Global Health and Medicine (NCGM). All experiments were conducted with prior approval from the Animal Experimentation Committee of NCGM. KYSE30/Vector cells and KYSE30/PITX1 cells (5.0 × 10^6^) were inoculated subcutaneously in the left and right flank of 8-week-old BALB/c nude mice, respectively. After 3 weeks, tumor size was measured with calipers. Tumor volume was determined by long (a) and short (b) diameters with height (c), calculated as volume = a × b × c × 3.14/6.

### Statistical analysis

Each tumor was classified based on tumor location and the pathologic tumor, lymph node, metastasis (pTMN) classification (7^th^ edition, 2009). Methylation of *PITX1* was compared by using the Mann-Whitney U test for age; the Fisher exact test for gender, tumor location, pT status, pN status, pM status, and disease stage. Survival curves were calculated by the Kaplan-Meier product-limit estimate method and then examined using the Gehan-Breslow-Wilcoxon procedure. The data were expressed as the mean ± SD, and the results were compared by paired or unpaired Student's *t*-test. Statistical analyses were made with the Prism 5 statistical program (GraphPad Software, Inc., La Jolla, CA). All tests were two-tailed, and *P* values < 0.05 were considered significant.

## Abbreviations

ESCC: esophageal squamous cell carcinoma
SAGE: serial analysis of gene expression
MeDIP: methyl-DNA immunoprecipitation
PITX1: paired-like homeodomain 1
PPL: periplakin
NGS: next-generation sequencing
5-aza-dC: 5-aza-2′-deoxycytidine
DEG: differentially expressed gene
DMR: differentially methylated region
TSS: transcriptional start site
TERT: telomerase reverse transcriptase
NF-κB: nuclear factor-κB
RASAL1: RAS protein activator like 1
MAPK: mitogen-activated protein kinase
